# DECODING EHR DATA: THE ROLE OF SOCIAL SUPPORT IN PRESSURE INJURIES AMONG HOMEBOUND OLDER ADULTS

**DOI:** 10.1093/geroni/igae098.4154

**Published:** 2024-12-31

**Authors:** Christine Chow, Yijung Kim, Lauren Bangerter, Karl De Jonge

**Affiliations:** Georgetown University Medical Center, Arlington, Virginia, United States; Medstar Health Research Institute, Hyattsville, Maryland, United States; Medstar Health, Hyattsville, Maryland, United States; MedStar Washington Hospital Center, Washington, District of Columbia, United States

## Abstract

Homebound older adults are increasingly susceptible to acquiring pressure injuries (PIs) due to a variety of pathological and physiological changes associated with aging. However, the impact of social risk factors of PIs among homebound older adults remains largely understudied. This research was conducted to explore how unstructured data via House Call clinical notes can provide insights of psychosocial factors that influence pressure ulcer conditions compared to formal diagnoses via ICD-10 codes. Using electronic health records and clinical notes (N= 1,770) from patients enrolled in a home-based primary care program (04/01/2021-03/31/2024), we evaluate the impact of a novel natural language process (NLP) algorithm to identify PIs among older adults (N= 664 patients, Mean age= 86.55, 78% female, 84% Black or African American). These clinical notes were analyzed using bigram analysis to identify patterns of psychosocial and other factors that influence documentation of pressure ulcers. Themes included known common risk factors for PIs (functional impairment, malnutrition, co-morbidities, frailty, and poor tissue perfusion). However, social support was a unique category identified with multiple associated bigram phrases, suggesting a strong relationship with PIs and underscores a need for further investigation. This study highlights the emerging potential of NLP methodology in clinical research for the systematic analysis of large volumes of clinical narratives, offering valuable insights from these interpersonal relationships. Furthermore, understanding social risk factors can contribute to the early identification of PIs, inform clinical practices, and help reduce the incidence of PIs in homebound older adults.

